# A New Technique for the Extraction of Arbuscular Mycorrhizae Fungal Spores from Rhizosphere

**DOI:** 10.3390/jof9080845

**Published:** 2023-08-14

**Authors:** Gökhan Boyno, Semra Demir, Younes Rezaee Danesh, Emre Demirer Durak, Rojbin Çevik, Beatrice Farda, Rihab Djebaili, Marika Pellegrini

**Affiliations:** 1 Department of Plant Protection, Faculty of Agriculture, Van Yuzuncu Yil University, Van 65090, Türkiye; gokhanboyno@yyu.edu.tr (G.B.); y.rdanesh@yahoo.comemredemirer@yyu.edu.tr (E.D.D.); rojbincevik63@gmail.com (R.Ç.); 2 Department of Plant Protection, Faculty of Agriculture, Urmia University, Urmia 5756151818, Iran; 3 Department of Life, Health and Environmental Sciences, University of L’Aquila, Coppito, 67100 L’Aquila, Italy; beatrice.farda@graduate.univaq.it (B.F.); rihab.djebaili@guest.univaq.it (R.D.)

**Keywords:** arbuscular mycorrhiza, spore bank dynamics, sustainable agriculture, new technique

## Abstract

Monitoring the dynamics of the spore bank of arbuscular mycorrhizal fungi (AMF) is essential for the sustainable management and protection of agroecosystems. The most common method for extracting AMF spores from soil is the wet-sieving technique (WST). However, this method has many disadvantages. In this study, we modified the WST using new approaches: the ultrasound wet-sieving technique (UWST) and the ultrasound centrifuge technique (UCT). We enumerated and compared the numbers and quality of spores obtained from WST, UWST, and UCT to validate the new modified techniques. We extracted AMF spores from the rhizospheres of different plants, including wheat (*Triticum aestivum* L.), bean (*Phaseolus vulgaris* L.), tomato (*Solanum lycopersicum* L.), pepper (*Piper nigrum* L.), parsley (*Petroselinum crispum* Mill.), and turfgrass (*Lolium perenne* L.) collected from the Van Lake basin, Turkey. The highest and lowest AMF spore numbers were observed in wheat and turfgrass rhizospheres. The UCT allowed for the extraction of the highest number of spores from all rhizospheres, followed by the UWST and WST. The UWST and WST allowed for the extraction of similar spore numbers from wheat, pepper, parsley, and turfgrass rhizospheres. Beyond the high extracted spore number, UCT was shown to be a fast and low-material-consuming approach. These findings demonstrate that the UCT can be used to efficiently extract AMF spores in future research.

## 1. Introduction

Arbuscular mycorrhizal fungi (AMF) are typical soil-inhabiting microorganisms forming a mutual symbiosis with more than 80% of land plants [1,2,3]. AMF are obligate symbiotic biotrophs, consuming within their life cycle lipids and plant photosynthesis products [4]. After the hyphal entrance into the root cortex, AMF complete their life cycle and sporulate using glucose as a carbon source [5]. At the same time, AMF improve the growth and development of plants, especially in agricultural soils [6,7]. AMF mobilize inert soil mineral nutrients and enhance the uptake of nutrients by plants. AMF also promote plant tolerance and resistance against various biotic and abiotic stresses, i.e., phytopathogen attacks [8,9], drought [10,11], and salt stress [12].

Spores ensure AMF survival and activity in agroecosystems. These structures are the active ingredients of AMF-based biofertilizers, and their extraction is a key step in many studies [13,14]. AMF spores in the soil can shift into dormant structures in response to adverse environmental changes [15]. Alterations of environmental conditions (e.g., temperature, humidity, availability of nutrients) trigger the creation of a dynamic spore bank and dormancy cycles (sporulation and germination) [14,15]. Spore bank dynamics are essential in AMF biodiversity, mycorrhizal associations, plant species growth and development, and ecosystem processes [15,16].

There are several studies devoted to the genetic diversity of AMF spores [17,18] and their ecology [19], physiology [20], and inoculum production [21]. In most studies, the assay of AMF spore extraction is a crucial step. One of the most significant challenges is the extraction of AMF spores without any unwanted particles (soil and plant debris). AMF spores are usually isolated by wet-sieving and centrifugation with a sucrose solution (WST) [22,23]. Some researchers have developed improved methods for spore extraction [24,25,26,27,28,29]. These methods mostly use a sieving step at 45 µm to extract AMF spores (50–750 µm and up to 1 mm) and exclude those of other fungi (2–10 µm range) [30]. However, there is the presence of multiple unwanted substances that are extracted together. Removing these interferents requires the utilization of fine-diameter mesh filters and leads to the wastage of water [28,29]. Sugar-based solutions are employed to improve the purity of the spore suspension. Nevertheless, these solutions have a detrimental effect on spore morphology [28]. Therefore, new methods must be developed to overcome the current limitations and disadvantages.

The aim of our study was to develop a new technique with improved performance for AMF spore extraction. Ultrasounds can change the physical properties of materials by specific frequencies of sound waves [31]. Therefore, we hypothesized that ultrasound use in extracting AMF spores could improve the standard WST protocol (UWST). This hypothesis was supported by previous studies that underlined the efficacy of ultrasounds in the extraction of AMF spores. For example, Satomura et al. optimized the extraction of AMF spores from clayey soil using ultrasonic extraction (30 s) and soil shaking (40 min) [32]. Moreover, utilizing ultrasounds and substituting sieves and filters with centrifugation, we set up a new technique to improve spore extraction (UCT). We determined and compared the number of AMF spores from the rhizospheres of different plant species using the WST, UWST, and UCT to test our hypothesis and validate the methods.

## 2. Materials and Methods

### 2.1. Site Description

**Figure 1:**
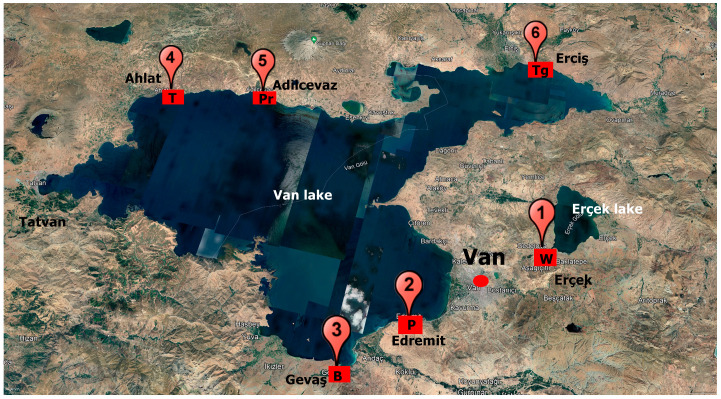
Sampling site locations: 1: wheat (W), 2: pepper (P), 3: bean (B), 4: tomato (T), 5: parsley (Pr), 6: turfgrass (Tg).

Rhizospheres of wheat (*Triticum aestivum* L.) (W), bean (*Phaseolus vulgaris* L.) (B), tomato (*Solanum lycopersicum* L.) (T), pepper (*Piper nigrum* L.) (P), parsley (*Petroselinum crispum* (Mill.)) (Pr), and turfgrass (*Lolium perenne* L.) (Tg) were collected at different Turkish productive landscapes (Figure 1). These plant species were selected based on their relevance to Turkish food and forage agriculture. Six sites around Lake Van in the highlands of eastern Anatolia were sampled based on the crop distribution and availability of farmers collaborating with Van Yuzuncu Yil University. The climate of the area is continental, with constant drought increases. The average annual low and high temperatures are 5.7 °C and 15.2 °C, respectively. The annual average rainfall is 310 mm. The precipitations mainly fall during the winter and spring seasons. The highest and lowest precipitations are in April and August, respectively. Monthly trends of temperature and precipitations are provided in Figures S1 and S2, respectively. The land use is agricultural and pastoral.

### 2.2. Rhizosphere Sampling

Ten plants of each species were randomly selected from the field. Plants and their rhizospheres were collected in a 20 cm^2^ by 30 cm deep sod during growth. The rhizosphere was collected by handshaking the plants and creating a homogenous composite sample. After air-drying, composite samples were passed through a 2 mm sieve and stored at +4 °C until analyzed.

### 2.3. Wet-Sieving Technique (WST)

**Figure 2:**
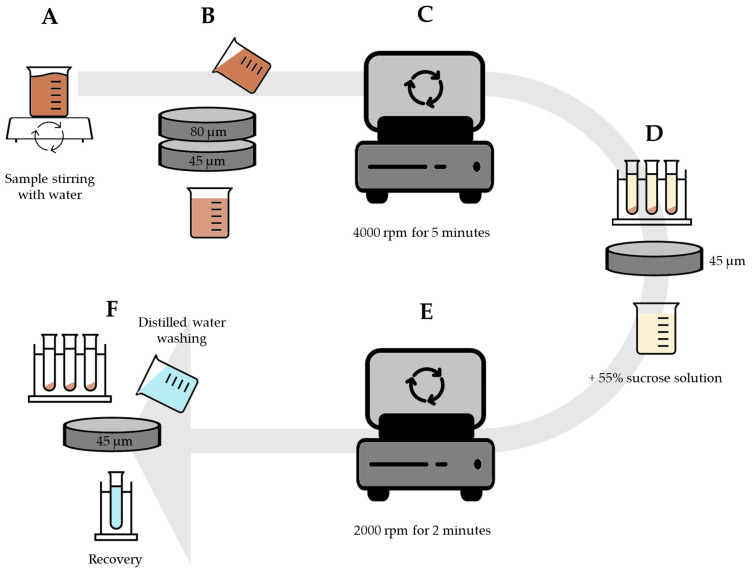
The process of extracting AMF spores using the wet sieving technique (WST). (**A**): Mixing soil sample with distilled water using magnetic stirrer; (**B**): Pouring the mixture through 80 µm and 45 µm sieves; (**C**): Centrifugation of liquid; (**D**): Pouring the supernatant through the 45 µm sieve and mixing with sucrose solution; (**E**): The second centrifugation; (**F**): Transferring the su-pernatant to 45 µm sieve, washing with tap water in new collection tube.

A total of 10 g of rhizosphere was mixed with 100 mL of distilled water using a magnetic stirrer for 1 min (Figure 2A). The mixture was poured through two sieves, first 80 µm and then 45 µm (Figure 2B). The resulting liquid was centrifuged at 4000 rpm for 5 min (Figure 2C) and the supernatant was poured back into the 45 µm sieve (Figure 2D). The sieve content was thoroughly mixed with a 55% sucrose solution and centrifuged at 2000 rpm for 2 min (Figure 2E). The supernatants obtained were transferred to a 45 µm sieve and washed thoroughly with tap water to remove the sucrose, then placed with 20 mL of water in a new collection tube (Figure 2F).

### 2.4. Ultrasound Wet-Sieving Technique (UWST)

**Figure 3:**
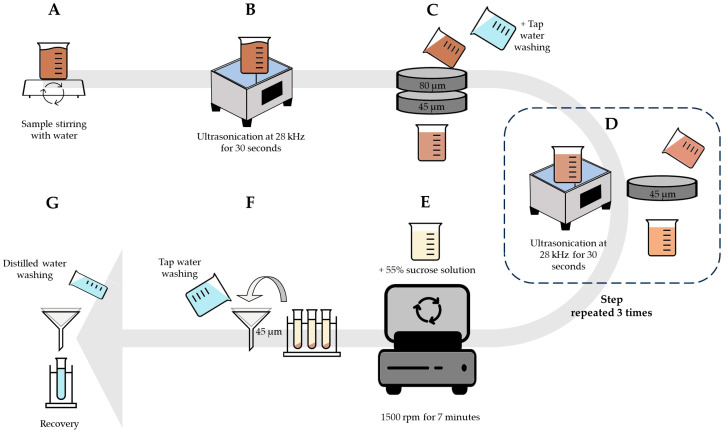
The process of extracting AMF spores using the ultrasound wet-sieving technique (UWST). (**A**): Mixing soil sample with distilled water using magnetic stirrer; (**B**): Treating the mixture using ultrasound; (**C**): Pouring the mixture through 80 µm and 45 µm sieves; (**D**): Treating the mixture using ultrasound and 45 µm sieving; (**E**): Adding sucrose solution and centrifugation; (**F**): Pouring the supernatant through the 45 µm filter paper and washing with tap water; (**G**): Washing the filter paper with distilled water and transferring to new collection tube.

A total of 1 g of rhizosphere was mixed with 20 mL of distilled water using a magnetic stirrer for 1 min (Figure 3A). The mixture was treated with an ultrasound at 28 kHz for 30 s (UltraClean12 Moodel, Hydraultrasonic Cop., Istanbul, Türkiye) (Figure 3B). After ultrasonication, the mixture was poured through two sieves (80 µm and 45 µm). The sieve content was washed in a new container (Figure 3C) and subjected to ultrasound (28 kHz for 30 s) and sieved at 45 µm three times (Figure 3D). The final sieve content was thoroughly mixed with a 55% sucrose solution and centrifuged at 1500 rpm for 7 min (Figure 3E). The supernatants were passed through 45 µm filter papers and washed thoroughly with water for sucrose removal (Figure 3F). The filter paper was washed with 20 mL of distilled water and transferred to a new collection tube (Figure 3G).

### 2.5. Ultrasound Centrifuge Technique (UCT)

**Figure 4:**
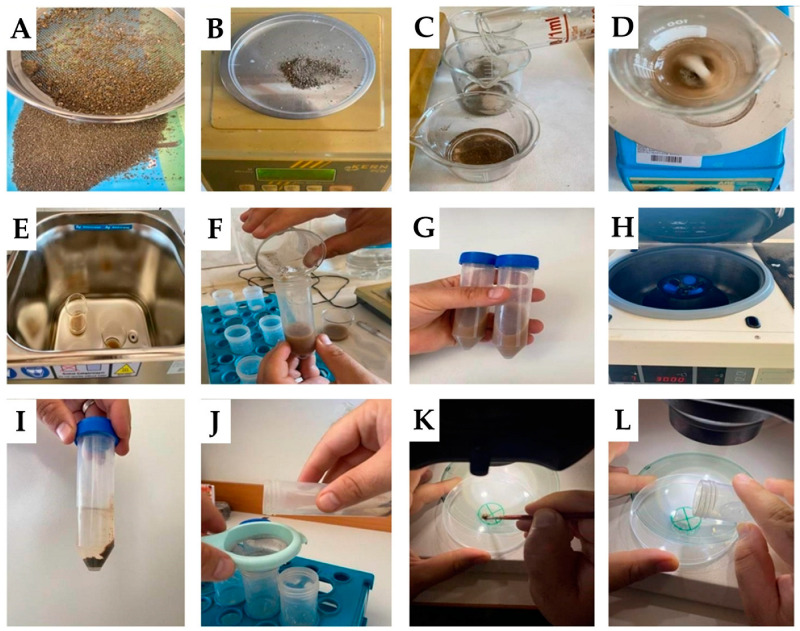
The steps of AMF spore extraction and enumeration using the ultrasound centrifuge technique (UCT). (**A**): Separation of rhizosphere sample from solid particles (using 2 mm sieve); (**B**): Weighing the sample; (**C**): Adding the sample to the beaker and transferring the distilled water; (**D**): Stirring the sample in a magnetic stirrer; (**E**): Treating the mixture with ultrasound; (**F**): Transferring the mixture into falcon tubes; (**G**): Mixing the mixture by hand; (**H**): Centrifugation of the mixture; (**I**): The supernatant and pellet of the mixture; (**J**): Transferring the supernatant through the strainer into new falcon tubes; (**K**): Using glycerin to the circle drawn for counting on the petri dishes; (**L**): Transferring the spore suspension to the drawn circle and counting it under the stereo microscope.

A total of 1 g of rhizosphere was mixed with 20 mL of distilled water using a magnetic stirrer at 4–5 rpm for 5 min (Figure 4A–D). The mixture was treated with an ultrasound at 28 kHz for 30 s (UltraClean12 Moodel, Hydraultrasonic Cop., Istanbul, Türkiye —Figure 4E). The mixture was then transferred to the falcon tube (Figure 4F,G) and centrifuged at 3000 rpm for 3 min (Figure 4H,I). The supernatant was passed through a 1 mm strainer and transferred to a new collection tube (Figure 4J).

### 2.6. AMF Spore Enumeration

**Figure 5:**
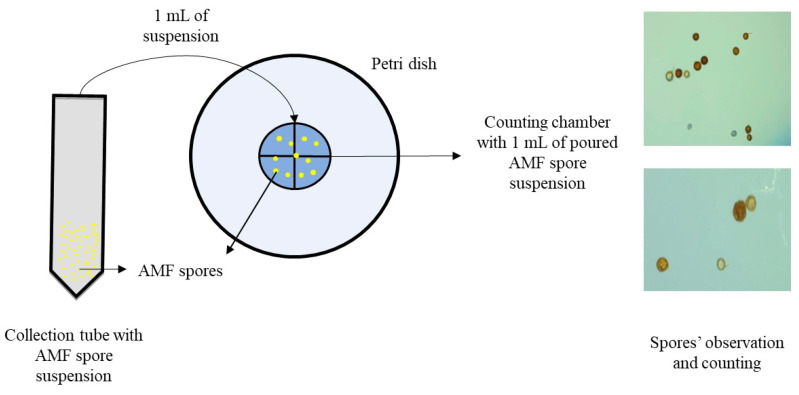
AMF spore counting.

A circle (2.5 cm diameter) divided into four equal parts was designed on the back side of a Petri dish (Figure 4K). One drop of glycerin (or lactic acid) was layered inside the circle using a fine paintbrush (Figure 4L). The presence of this layer slowed down the fluid movement and facilitated spore enumeration. A total of 1 mL of the AMF spore suspensions obtained after using WST, UWST, and UCT was placed onto the center of the circle for further spore counting (Figure 5).

The number of AMF spores in 1 g of the rhizosphere was calculated under a stereo microscope (40× magnification) according to the following formula:

$$\mathrm{TSN}\;=\left[\mathrm{SN}\;\times \;W\;\right]÷S$$

- TSN: Total AMF spore numbers in 1 g of rhizosphere
- SN: AMF spore numbers in 1 mL of spore suspension
- W: Amount of water used (mL)
- S: Amount of soil used (g)

### 2.7. Statistical Analysis

The extractions were carried out using a factorial experiment based on a completely random design (CRD). The studied factors were the extraction techniques (WST, UMT, and NMT) and plant species (W, B, T, P, Pr, and Tg). Each treatment had three replications (3 × 6 × 3 = 54 experimental units). A two-way unbalanced analysis of variance (ANOVA) was applied to test the effect of the extraction techniques, plant species, and the interaction between them on AMF spore numbers. The means were compared and grouped based on Duncan’s multiple range test (*p* < 0.01). All statistical analyses were carried out by SPSS software version 26 (IBM Corp., Armonk, NY, USA).

## 3. Results

A comparison of all techniques (Table 1) showed that the UCT method had a shorter duration than the other two techniques (WST and UWST). The two-way analysis of variance (Table 2) showed that both factors investigated (plant species and extraction technique), as well as the interaction between them, significantly influenced the AMF spore count (*p* < 0.01).

**Figure 6:**
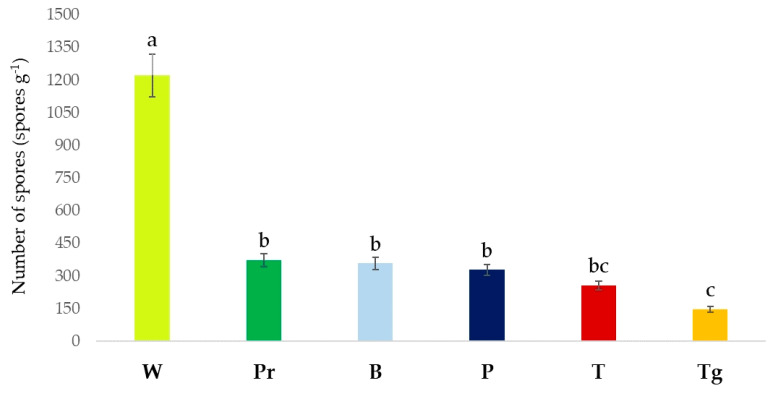
The AMF spore numbers estimated in the rhizosphere of different plants. W, *Triticum aestivum*; B, *Phaseolus vulgaris*; T, *Solanum lycopersicum;* P, *Piper nigrum*; Pr, *Petroselinum crispum*; Tg, *Lolium perenne*. Means followed by the same case letter (a–c) are not significantly different according to the Duncan multiple range test (*p* > 0.01).

Among the plant species, the highest number of AMF spores was counted in the rhizosphere of wheat (*p* < 0.01), with 1222 spores g^−1^, followed by parsley, bean, pepper, and tomato. The AMF spore numbers did not significantly differ in the rhizosphere of parsley, bean, and pepper (*p* > 0.01). The lowest AMF spore number (147 spores g^−1^, *p* < 0.01) was determined in the turfgrass rhizosphere, which had no significant difference from tomato (*p* > 0.01) (Figure 6).

**Figure 7:**
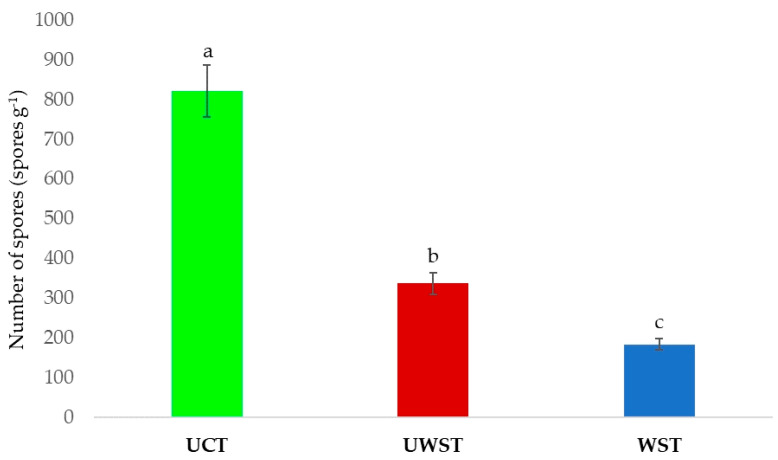
AMF spore numbers estimated using different extraction techniques. Means followed by the same case letter (a–c) are not significantly different according to the Duncan multiple range test (*p* > 0.01).

Irrespective of the plant rhizosphere effect, the highest average number of spores was obtained using the UCT (822 spores g^−1^, *p* < 0.01), followed by the UWST (337 spores g^−1^, *p* < 0.01). The lowest number of spores was obtained using the WST (184 spores g^−1^, *p* < 0.01) (Figure 7).

**Figure 8:**
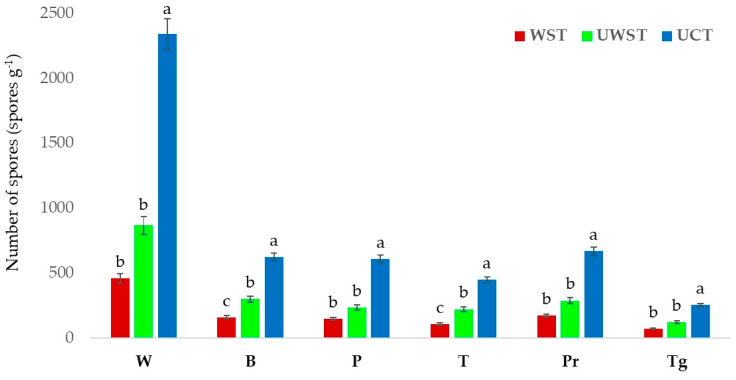
AMF spore numbers estimated in rhizospheres under different plant species using WST, UWST, and UCT. Means followed by the same case letter (a–c) are not significantly different according to the Duncan multiple range test (*p* > 0.01).

In the rhizosphere of all tested plants, the highest fungal spore count was obtained using the UCT. No significant differences were observed among W, P, Pr, or Tr (*p* > 0.01) (Figure 8).

## 4. Discussion

The current study compared the newly modified techniques, the UWST and UCT, with the WST—the standard method used to extract AMF spores. The extraction techniques used had a significant impact on the total number of AMF spores extracted. In all plant rhizosphere samples, the highest number of AMF spores was obtained using the UCT, followed by the UWST and WST. The lower yields recorded for the WST and UWST were probably associated with losing some spores during the sieving steps. Some spores can be blocked from passing through the top sieve since they are still attached to soil particles or fungal hyphae. Some large spores and sporocarps may be lost passing through the sieves. Therefore, these spores may not be considered in enumeration, affecting the estimated number of spores. In the proposed new modified technique, using an ultrasound induced the separation of AMF spores from soil particles so that they could be observed clearly. In addition, fungal spores, particularly those that had big sizes and sporocarps, could be effortlessly extracted without the need for sieves. This method proved to be highly effective, leading to a substantial increase in the number of fungal spores that could be extracted. The ultrasound’s ability to separate coarse soil particles from fungal spores (and from each other) has already been reported for frequencies higher than 50 kHz [32,33].

The results showed a significant difference in AMF spore numbers among different plant rhizosphere samples. This aspect is not surprising considering the ability of plants to shape the composition and richness of rhizosphere microbial communities [14,34,35]. Differences could also be observed within the same plant species based on ecotype, genotype, stage of plant development, root architectures, and root exudates [36]. Other soil characteristics, such as the type and availability of nutrients and cation exchange capacity (an indicator of soil structure, pH, and the content of nutrients that plants can absorb), can also impact the number of AMF spores. Moreover, AMF sporulation patterns are influenced by the soil’s pH and texture [37]. The overall composition of the AMF spores is also influenced by temperature increases and nitrogen addition [38]. Due to the influence of these specific plant-related factors, explaining the variation in AMF spore numbers observed in wheat and turfgrass samples is challenging.

The UCT has several advantages over the other two methods. These include saving time, low water use, recovering spores with a preserved structure, and extracting more spores from low amounts of rhizosphere samples. Time is a significant and critical factor in laboratory experiments. In both the WST and the UWST, it is necessary to spend more time extracting fungal spores due to using sieves and centrifugations. In addition, the WST needs at least 10 g of a rhizosphere sample for fungal spore extraction [14,39,40]. On the other hand, the UCT allows for the efficient processing of multiple samples of small amounts (1 g) in a relatively short time (around 8 min for a minimum of six samples). In the WST and UWST, a lot of water is used during the various washing phases. Conversely, UCT uses only 20 mL of water, ensuring the protection of this essential resource.

Beyond economic and environmental sustainability, less water use preserves the quality of spores, whose structures are damaged by continuous washing steps [28]. Spore integrity preservation is essential, especially for morphological identification and the further characterization of AMF spores. Given the high spore integrity obtained, UCT is a valid extraction technique to be used in identification studies. The spore suspensions obtained with the UCT can be used to prepare microscopic slights and studied with microscopic techniques [41,42]. Since AMF morphological identification is based on the morphological features of spores (e.g., size, color, wall structure), the preservation of their integrity is crucial [43].

## 5. Conclusions

In this research, we proposed and developed a modified technique for extracting AMF spores. It detects a higher spore number, shortens the extraction duration, and consumes less water, obtaining more undamaged and clean spores than other methods. Further research is required to assess the efficacy of the UCT in extracting AMF spores from the rhizospheres of various plant species obtained from diverse pedoclimatic environments. Given the positive outcomes for other plant rhizospheres, we assume that the method and formula developed here can be efficiently used worldwide. The UCT has the potential to emerge as an innovative, cost-effective, and environmentally friendly method for extracting AMF spores. The technique could be applied for scientific studies and by enterprises and laboratories to control AMF-based biostimulant formulations. Using a faster and more efficient technique in the AMF extraction process can improve AMF-based products. Moreover, the possibility of applying a faster and more efficient technique in evaluating spore bank dynamics can support the commercialization of high-quality products. These aspects are particularly relevant in the current scenario of global food production, agricultural sustainability, and environmental protection. They are also in line with the 2030 United Nations Agenda goals for Sustainable Development.

## Figures and Tables

**Table 1:** Wet-sieving technique (WST), ultrasound wet-sieving technique (UWST), and ultrasound centrifuge technique (UCT) details.

Techniques	Magnetic Stirrer	45 and 80 µm Sieves *	Ultrasound (Total)	Centrifuge (Total)	Filtration Through Filter Papers *	45 µm Sieve *	Total Time
WST	1 min	~3 min	-	7 min	-	~2 min	13 min
UWST	1 min	~3 min	1.5 min	7 min	~3 min	-	15.5 min
UCT	5 min	-	0.5 min	3 min	-	-	8.5 min

*: These values were measured during sample processing.

**Table 2:** Results of two-way unbalanced analysis of variance (ANOVA) on the effects of plant species (A), extraction technique (B), and their interactions (A × B) on AMF spore counts.

	Degrees of Freedom	Mean Square
A	5	1,356,710.163 **
B	2	1,998,088.963 **
A × B	10	322,316.430 **
Error	36	
Total	53	

A, plant species; B, extraction technique; **: Significant at *p* < 0.01. R^2^ = 98.9%.

## Data Availability

The data that support the findings of this study are available upon request from the corresponding author.

## Supplementary Materials

The following supporting information can be downloaded at https://www.mdpi.com/article/10.3390/jof9080845/s1: Figure S1: Average High and Low Temperature recorded at sampling sites. Data retrieved from https://weatherspark.com/; Figure S2: Average Monthly Rainfall recorded at sampling sites. Data retrieved from https://weatherspark.com/.
