# Supplementary material for: A New Technique for the Extraction of Arbuscular Mycorrhizae Fungal Spores from Rhizosphere
Source: J Fungi (Basel). 2023 Aug 14;9(8):845. doi: 10.3390/jof9080845 (PMC10455966; doi:10.3390/jof9080845)
Supplement: Supplementary file 1 [file jof-09-00845-s001.zip › jof-2478660-supplementary.pdf]

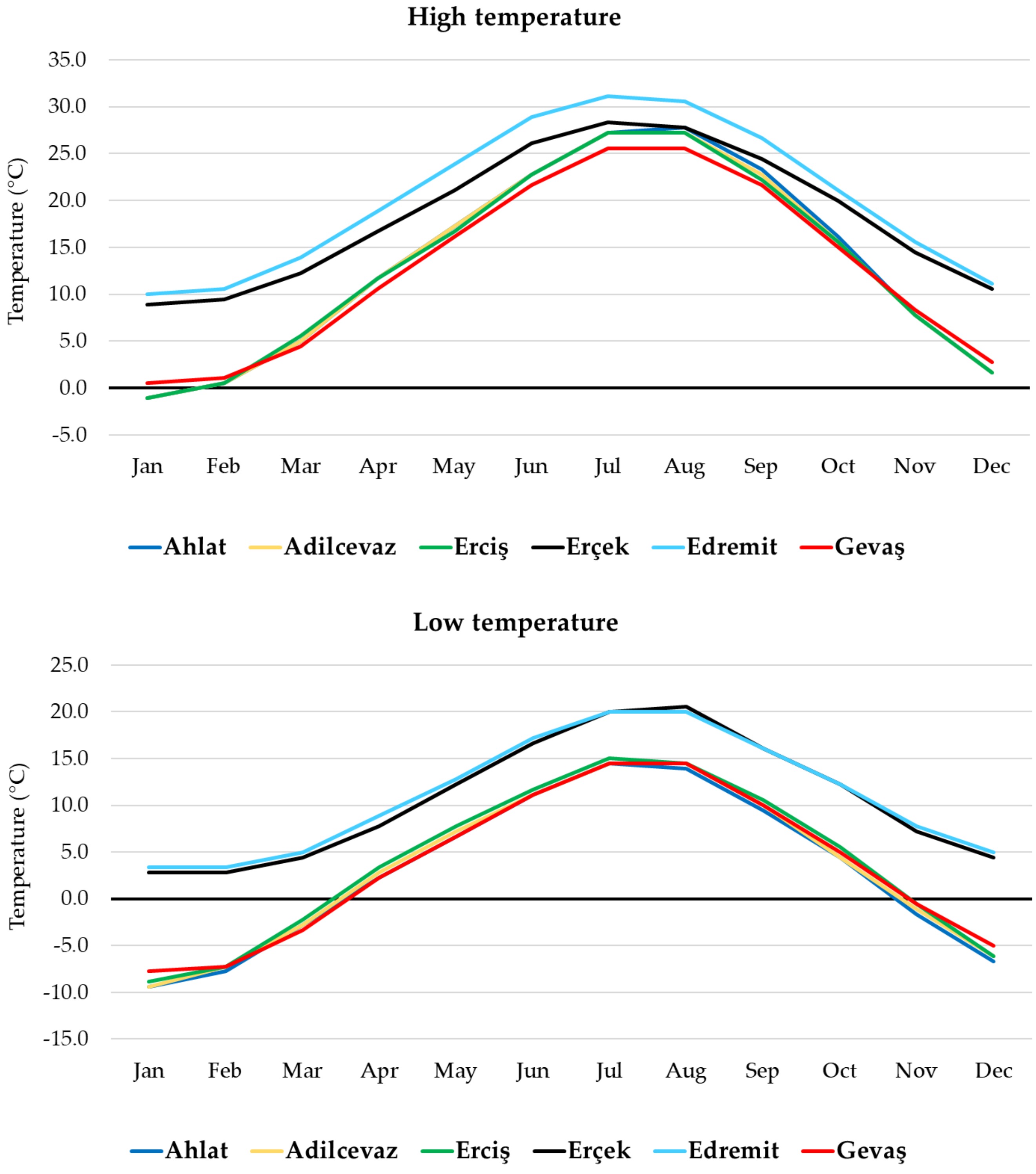

Figure S1. Average High and Low Temperature recorded at sampling sites. Data retrieved from https://weatherspark.com/

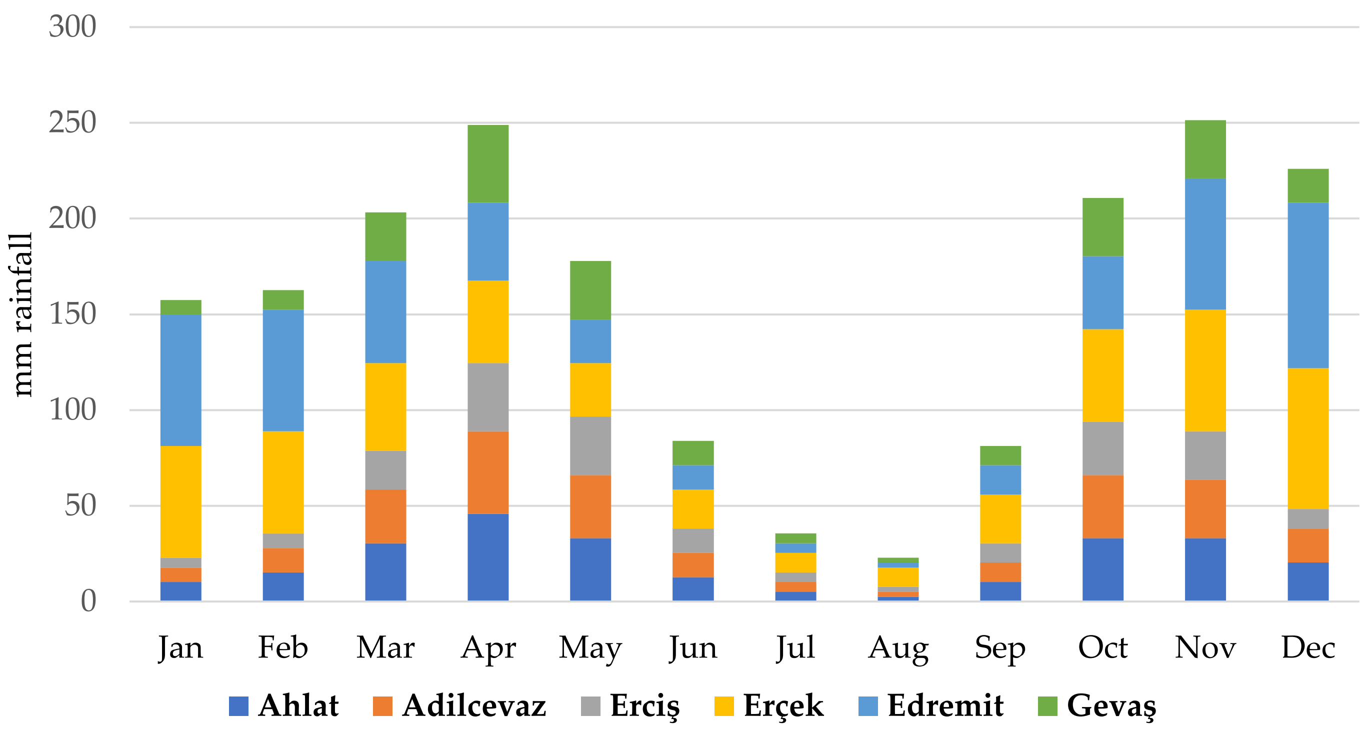

Figure S2. Average Monthly Rainfall recorded at sampling sites. Data retrieved from https://weatherspark.com/
